# Pituitary hypophysitis in granulomatosis with polyangiitis (GPA): a case series

**DOI:** 10.1007/s11102-023-01378-5

**Published:** 2024-01-31

**Authors:** Majid Alameri, Abdulla Alnuaimi, Niamh M. Martin, Karim Meeran, Anastasia Gontsarova, Tara D. Barwick, Spencer Ellis, Stephen McAdoo, James Tomlinson, Florian Wernig

**Affiliations:** 1 Department of Endocrinology, Imperial College Healthcare NHS Trust, London, UK; 2 Department of Radiology, Imperial College Healthcare NHS Trust, London, UK; 3 Department of Surgery and Cancer, Imperial College London, London, UK; 4 Department of Rheumatology, East and North Hertfordshire NHS Trust, Stevenage, UK; 5 Department of Renal Medicine, Imperial College Healthcare NHS Trust, London, UK; 6 Department of Immunology & Inflammation, Imperial College London, London, UK

**Keywords:** Granulomatosis with polyangiitis, ANCA-associated vasculitis, Rituximab, Hypophysitis

## Abstract

Granulomatosis with polyangiitis (GPA) rarely involves the pituitary gland. Pituitary involvement has been reported in ~ 1% of all cases of GPA. Most commonly, pituitary swelling and inflammation results in symptoms due to pituitary mass effect and arginine vasopressin deficiency. To date, there are no pituitary-specific treatment guidelines for this rare condition. We present three patients with GPA-related hypophysitis highlighting the spectrum of pituitary involvement. All three patients were successfully treated with immunosuppressive regimens that included rituximab (RTX). Following remission induction with high-dose glucocorticoids, patients received 6 monthly RTX for remission maintenance. RTX was well tolerated without significant side effects.

## Introduction

Granulomatosis with polyangiitis (GPA) is a necrotizing vasculitis of predominantly small to medium-sized arteries comprising a large spectrum of variable organ involvement and disease severity. One of the pathological hallmarks is the strong association with anti-proteinase 3 anti-neutrophil cytoplasmic antibodies (PR3-ANCA); GPA is part of the group of ANCA-associated vasculitides (AAV) [1]. The upper and lower respiratory tract as well as kidneys are the most commonly affected organs (70–100% and ~ 70% respectively) [2]. GPA appears to involve the pituitary gland in approximately 1% of all cases [3, 4]. Pituitary GPA commonly presents due to a pituitary mass effect resulting in headache, visual disturbance as well as anterior and posterior pituitary hormone deficiencies. Arginine Vasopressin deficiency (AVPD) due to the infiltration of the posterior pituitary appears to be the most frequent hormone deficiency (67–78%) followed by secondary hypothyroidism (50–71%) and ACTH deficiency (11–12.5%) [3–5]. Proposed mechanisms include vasculitis of central nervous system blood vessels and granuloma formation within the pituitary or invasion from nearby anatomic areas such as the paranasal sinuses or orbits [6]. Current treatment algorithms for systemic GPA recommend remission induction with high-dose glucocorticoids together with cyclophosphamide or rituximab (RTX) followed by a less immunosuppressive maintenance therapy of RTX and/or conventional steroid-sparing agents [7]. RTX appears to have been most effective in maintaining remssion and it has been proposed to evaluate the combination of cyclophosphamide with RTX versus RTX only [7–9]. Pituitary GPA can often present as a late manifestation following wider systemic GPA and its most effective management is uncertain. Here, we present three cases of pituitary GPA successfully treated with glucocorticoids and RTX maintenance therapy.

## Patients and methods

Patients were identified and diagnosed with pituitary GPA by a multidisciplinary pituitary review panel at Imperial College Healthcare NHS Trust between November 2019 and August 2022 (Table 1). Treatment decisions were guided by a multidisciplinary pituitary and vasculitis panel and reviewed at regular intervals. Clinical, radiological and laboratory data were collected retrospectively using the hospital’s electronic health records (Cerner Millenium, Oracle Corporation, Austin Texas).

**Table 1:** Patient characteristics, presenting symptoms, visual fields, immunology and endocrine dysfunction at presentation

Patient	Age at initial presentation of GPA	Sex	Ethnicity	Systemic GPA involvement	Presenting hypophysitis symptoms	Time of onset of GPA to hypophysitis presentation	Visual field	C-ANCA	PR3	Endocrine dysfunction at presentation
**1**	52	F	White British	Lungs and sinuses; no renal involvement	Headaches, polyuria and polydipsia; visual problems	24 months	Bitemporal hemianopia	Positive	22.00 IU/L	Central AVPD, secondary hypoadrenalism; secondary hypothyroidism, and secondary hypogonadism
**2**	38	F	White British	Mild sinusitis and intermittent epistaxis	Recurrent headaches, epistaxis, polyuria and polydipsia	Same time as onset of symptoms	Minor visual field defect only	Positive	13.00 IU/L	Central AVPD; transient secondary hypoadrenalism; could not assess gonadal axis (contraceptive implant)
**3**	48	M	Asian (Indian)	Sinusitis and intermittent epistaxis; saddle nose deformity; no lung or renal involvement	Headaches, pneumonia, polyuria and polydipsia	Same time as onset of symptoms	bitemporal quadrantanopia	Positive	4.30 IU/L	Central AVPD; secondary hypoadrenalism; secondary hypothyroidism; secondary hypogonadism

*GPA* granulomatosis with polyangiitis, *AVPD* arginine vasopressin deficiency

Review of the literature was carried out by performing a Medline search using the terms ‘Granulomatosis with polyangiitis’ or ‘Wegener’ and ‘pituitary’ or ‘hypophysitis’ to identify published articles. Only articles published in English language and relevant information and clinical summaries were included.

### Case 1

**Fig. 1:**
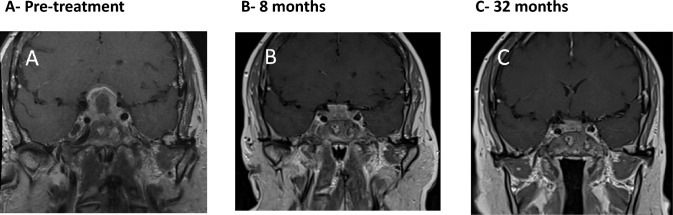
Coronal post-contrast T1W1 MR images of the pituitary. **A** Pre-treatment pituitary MRI scan showing a large enhancing centrally necrotic sellar and suprasellar lesion which elevates and compresses the optic chiasm. **B** Image 8 months following high dose GC treatment, MR images of the pituitary showing the initial response to treatment with size reduction of the sellar/suprasellar lesion. There is still minimal contact with the optic chiasm. **C** Pituitary MRI at 32 months, showing improvement and decrease in size of pituitary mass

A 55-year-old female was diagnosed with PR3-ANCA-positive GPA with pulmonary involvement, a cavitating lung lesion, which responded well to induction treatment with immunosuppressants and glucocorticoids (six pulses of pulsed intravenous cyclophosphamides per EUVAS ANCA-Vasculitis protocol of 15 mg/kg, and variable doses of prednisolone/methylprednisolone). After induction therapy, she received mycophenolate mofetil (MMF) and low dose oral prednisolone and remained well in clinical remission. 28 months after first presentation, she developed blurred vision, bitemporal hemianopia and clinical symptoms of AVPD, which was confirmed by a water deprivation test. Pituitary MRI showed a large sellar mass with suprasellar extension and inflammatory changes suggestive of pituitary GPA. There was peripheral enhancement with central necrosis and elevation and compression of the optic chiasm. Furthermore, there was high hypothalamic signal as well as high signal within the optic chiasm and optic tracts. Due to the size of the lesion, the pituitary gland and the pituitary stalk could not be identified. (Fig. 1A). She was started on high-dose prednisolone (60 mg) with a plan to taper the dose gradually. Pituitary MRI was repeated 8 weeks later after starting high-dose prednisolone and revealed reduction in the size of the sellar and suprasellar mass lesion which contacted, but no longer compressed the optic chiasm and there was resolution of the hypothalamic high signal. Following initial remission induction with high-dose steroids, she received rituximab (RTX; 2 × 1 g doses, 2 weeks apart) together with cyclophosphamide (5 pulses). Humphrey’s visual field perimetry showed significant improvement of her bitemporal hemianopia. RTX treatment led to expected B-cell depletion and decrease in PR3-ANCA levels (from 22 to 11 IU/mL [0–1.9]). Whilst prednisolone was gradually reduced to a maintenance dose of 5 mg, her condition and vision remained stable. Blood tests showed that PR3-ANCA continued to further decrease to 6.7 IU/mL with continued B-cell depletion. A repeat pituitary MRI scan 6 months later showed some increase in the size of the sellar and suprasellar pituitary mass lesion and prednisolone was therefore temporarily increased to 30 mg once daily. Two months later, pituitary MRI showed reduction in the size of the pituitary lesion from 17 to 12 mm in maximal cranio-caudal dimension indicating response to treatment (Fig. 1B**)**. She received further 10 to 12-monthly single doses of RTX with further clinical, biochemical and radiological improvement. She is currently being maintained on 6-monthly RTX infusions, with no concurrent therapeutic glucocorticoids and remains in full remission with undetectable PR3-ANCA 40 months after the initial pituitary presentation (Fig. 1C). She had persistent AVPD and remains on desmopressin (DDAVP) replacement therapy. She also remains on prednisolone replacement (4 mg daily) and levothyroxine replacement for secondary hypoadrenalism and secondary hypothyroidism respectively.

### Case 2

**Fig. 2:**
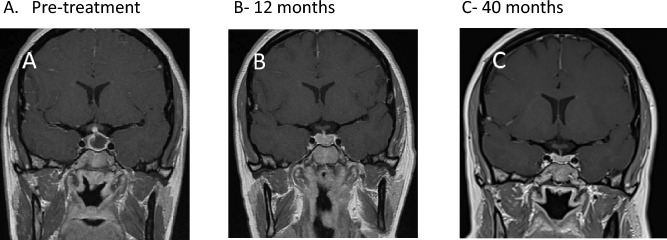
Coronal post-contrast T1W MR images of the pituitary. **A** Pre-treatment scan MRI pituitary showing a cyst-like peripherally enhancing pituitary lesion and a thickened pituitary stalk. There is distortion of the optic chiasm, but no compression. **B** Image following 6 months of treatment, MRI pituitary showing minimal residual enlargement of the pituitary gland. Resolution of the cyst-like component. Normal appearing pituitary stalk and optic chiasm. **C** 40 months of treatment, image showing radiologically normal appearing pituitary

A 38-year-old female presented with history of recurrent nosebleeds, severe headaches and a left visual field defect. Initial baseline investigations were suspicious of possible secondary adrenal insufficiency. Pituitary MRI showed a sellar heterogeneous cystic lesion of 14 mm in maximal cranio-caudal dimension with peripheral enhancement and a thickened stalk contacting and displacing the optic chiasm and hypothalamic enhancement (Fig. 2A), suggestive of an inflammatory aetiology. She started hydrocortisone replacement (20 and 10 mg daily), which led to immediate improvement in her symptoms, but she then developed cranial AVPD, and was started on DDAVP. A subsequent serological autoimmune/ hypophysitis screen revealed elevated PR3-ANCA (13 IU/mL [0–1.9]), suggestive of GPA-related hypophysitis. Following review by both pituitary and vasculitis multidisciplinary teams, it was felt that a diagnostic pituitary biopsy was not required and remission-induction with high-dose prednisolone was started. Her glucocorticoid dose was gradually reduced over a 6 month period and she received RTX (2 × 1 g doses, 2 weeks apart) for remission maintenance 6 months after her initial presentation. Six months later, pituitary MRI showed collapse of the cystic component related to the anterior pituitary gland and the infundibulum was not thickened (Fig. 2B). PR3-ANCA were undetectable, there was a continued excellent clinical and radiological response and she could be weaned from glucocorticoids (Fig. 2C). She remains on DDAVP for persistent AVPD. She has since received further 6-monthly RTX maintenance.

### Case 3

**Fig. 3:**
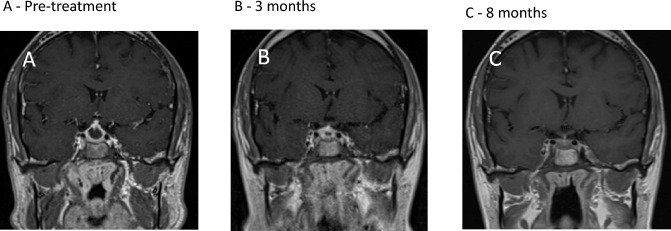
Coronal post-contrast T1W MR images of the pituitary. **A** Image at presentation showing an enlarged pituitary gland with peripheral enhancement and lack of enhancement centrally. The dome of the lesion abuts the optic chiasm. There is no optic chiasm compression. **B** Image done 3 months following remission induction with GC treatment showing reduction in size of the pituitary lesion with a small focus of a residual non-enhancing area centrally. **C** Follow up image at 8 months after treatment showing normal looking pituitary gland

A 48-year-old Asian male presented with a 4-week history of headaches, cough and recurrent nose bleeds. Clinical examination noted a saddle nose deformity. Chest X-ray confirmed pneumonia and he was treated empirically with a course of antibiotics. His cough improved and a repeat chest X-ray showed resolution of the infective changes without any other radiological lung abnormality. However, his headaches did not improve. Subsequent brain MRI revealed a sellar and suprasellar centrally cystic lesion with peripheral enhancement and thickening of the pituitary stalk. The lesion was noted to be in contact with optic chiasm causing mild upward displacement and measured 16 mm in maximal cranio-caudal dimension (Fig. 3A). Visual fields showed a bitemporal quadrantanopia. Pituitary hormonal profile showed secondary adrenal insufficiency, secondary hypothyroidism and secondary hypogonadism. Glucocorticoid (3 mg of prednisolone) and thyroid hormone replacement was started and further investigations for suspected autoimmune related inflammatory hypophysitis were carried out. c-ANCA was weakly positive and PR3-ANCA were elevated at 7.30 IU/mL [< 1.9]. CT head showed sinusitis. A repeat chest X-ray was normal. Following review by both a pituitary and a vasculitis multidisciplinary panel, a tapering course of prednisolone (starting at 60 mg daily) was commenced. Whilst on high-dose prednisolone, his headaches improved significantly, but he developed cranial AVPD requiring DDAVP replacement therapy. Pituitary MRI 3 months after the initial presentation showed significant improvement of the cystic component of the lesion (now measuring 10 mm in maximal cranio-caudal dimension) with improved shape and thickness of the infundibulum. There was no longer contact with the optic chiasm (Fig. 3B). Four months after his initial presentation, he received RTX (2 × 1 g doses, 2 weeks apart). Nine months after his initial presentation, he remains on maintenance prednisolone replacement only (3 mg daily) and his pituitary appearance had almost normalized (Fig. 3C) His PR3-ANCA remains undetectable. He continues on DDAVP for persistent AVPD. He remains under regular clinical follow up with a plan to continue 6-monthly RTX infusions for remission maintenance and periodic pituitary MRI surveillance.

## Literature review and discussion

We describe three patients representing a spectrum of pituitary GPA and variable degrees of systemic involvement. All three patients had disease features in keeping with the updated classification criteria for GPA recently endorsed by the American College of Rheumatology/European League Against Rheumatism (ACR/EULAR) 2022 [10]. There is no consensus regarding the diagnostic criteria to confirm pituitary involvement in GPA. GPA-related hypophysitis is commonly diagnosed in patients who present with pituitary-related hormonal dysfunction or visual defects in the presence of positivity for serological markers, in particular PR3-ANCA, with a history of GPA that can involve various organs including kidneys, lungs and sinuses. A large multicentre retrospective study of 819 patients with GPA reported 9 (1.1%) had pituitary involvement, 8 of which presented late after GPA initially manifested in other organs [11]. However, isolated pituitary GPA without systemic involvement has been reported [12]. A follow-up study of progression of GPA limited to the head and neck in 24 patients with an average follow-up of 6.8 years, showed that the disease spread to additional sites in more than half of the cohort (n = 14), but only 2 of 13 patients with disease initially limited to the head and neck developed pulmonary disease, and none developed renal disease [13]. Furthermore, patients with GPA-related hypophysitis may be negative for c-ANCA. 50–70% of patients with localised GPA may have negative ANCA testing, whereas 80–95% with systemic disease are positive for c-ANCA antibodies [10–12]. Pituitary biopsy does not routinely need to be undertaken if there is a clear clinical context of serological and systemic evidence of GPA. Confirmatory pituitary biopsy is required if the clinical scenario does not meet the criteria to diagnose GPA and if other aetiologies of a pituitary mass need to be ruled out [14–16]. Granulomatous inflammation and inflammatory infiltrates are typically found but are often non-specific in biopsy samples of pituitary tissue with GPA [14, 17].

Radiological findings of pituitary GPA have been described in case studies and case series and can be variable according to the central nervous system (CNS) structure(s) involved [3, 14]. Gadolinium-enhanced pituitary MRI is the optimum method for radiological assessment of the pituitary. Kapoor et al. reported that the radiological finding of a sellar mass with peripheral enhancement, central cystic changes and pituitary stalk compression were most commonly observed in patients with pituitary GPA. Other reports described radiological features of an enlarged gland with either heterogeneous or homogeneous enhancement [3, 15]. In addition, there is increased enhancement and thickening of the infundibulum, especially its superior portion [18]. Furthermore, a loss of the hyperintense signal in the posterior pituitary on T1-weighted sequences, explained by decreased AVP content in the posterior pituitary, was reported to be strongly correlated with the clinical manifestation of cranial AVPD in patients with GPA [3, 14, 15]. Importantly, normal MRI findings do not necessarily exclude pituitary involvement and anterior or posterior pituitary hormone dysfunction may be the only manifestation [4].

Given its rarity, there no studies of optimum immunosuppressive strategies in GPA-related hypophysitis, and specific recommendations are lacking in current international consensus guidelines (e.g., ACR, KDIGO, EULAR) [7, 19, 20]. Nor is this disease feature specifically considered within the spectrum of CNS or endocrine disease evaluated in commonly used disease assessment tools such as the Birmingham Vasculitis Activity Score (BVAS) [21]. Given the risk of pituitary failure, we suggest hypophysitis is regarded as a life-threatening disease complication warranting intensive treatment. We propose remission induction with glucocorticoids (prednisolone 60 mg daily followed by a weekly reduction to 45 mg, 30 mg, 25 mg and 20 mg and thereafter to be reduced by 5 mg every 2 weeks). We also propose to start RTX early initially 2 × 1 g doses 2 weeks apart and 1 g 6 monthly thereafter to achieve B-cell depletion.

In the published literature, the combination of cyclophosphamide and high-dose glucocorticoids has been used most frequently, and historically was shown to reduce 1-year mortality from 80% to 10–20% in patients with systemic GPA [9, 22]. However, toxicity of cyclophosphamide and the high relapse rate remain a challenge. The past decade has seen increasing use of RTX, a B lymphocyte-depleting monoclonal anti-CD20 antibody targeting B lymphocytes, including those producing ANCAs, both for remission induction and maintenance therapy. The landmark Rituximab in ANCA-Associated Vasculitis (RAVE) trial showed that treatment with RTX and glucocorticoids was equivalent to the standard combination of cyclophosphamide with glucocorticoids for attainment of disease remission at 6 months [23]. RTX is now recommended as a potential alternative to cyclophosphamide as first line therapy in current international consensus guidelines, although specific data on rare disease manifestations such as hypophysitis are lacking. Promisingly, a recent case series of 11 patients with CNS involvement of ANCA-associated vasculitis by Krishna et al. showed that RTX was as effective as cyclophosphamide in remission induction in patients with CNS involvement of GPA [24]. The use of cyclophosphamide and rituximab in combination has been studied in one randomised trial and several uncontrolled series, an approach that may facilitate rapid disease control and glucocorticoid-sparing, which was used successfully in one of our patients [25–28]. A regimen using glucocorticoid and RTX alone might be more suitable for patients where pituitary involvement is the predominant manifestation, but larger studies will be required to confirm our observational experience [29].

Regarding remission-maintenance, several studies have confirmed the superiority of regular rituximab infusions to conventional disease-modifying immunosuppressants, particularly in those with relapsing disease, administered either at fixed 4–6 monthly intervals or tailored to circulating B lymphocyte or ANCA levels, and this approach is now recommended depending on availability of rituximab [22, 24, 30]. Novel therapies for AAV, including GPA, include avacopan, a C5a-receptor antagonist that has shown efficacy as a glucocorticoid sparing agent, when used alongside rituximab or cyclophosphamide, in a recent large randomised controlled trial [31]. Avacopan was recently approved for treatment of AAV in the UK, although specific data regarding treatment of rare disease manifestations such as hypophysitis are lacking.

All our patients received combination therapies, including high-dose glucocorticoids with biological and/or immunosuppressant drugs (RTX, methotrexate, mycophenolate mofetil and cyclophosphamide), depending on the severity of pituitary GPA and the presence of other systemic manifestations (Table 2). In all cases, there was radiological evidence of GPA-related hypophysitis with excellent pituitary response to therapy.

**Table 2:** Duration of follow up, treatment regimen, radiological and biochemical pituitary response and systemic involvement of GPA

Patient	Duration of follow up	Treatment regimen	Pituitary imaging	Pituitary function	Systemic involvement
1	32 months	Remission induction with prednisolone, cyclophosphamide (5 2-weekly pulses of 500 mg) and RTX (2 × 1 g 2 weeks apart) Remission maintenance with 6 monthly RTX (1 g)	At presentation, large, enhancing and centrally necrotic sellar and suprasellar mass which elevates and compresses the optic chiasm; full resolution of radiological pituitary abnormalities	Panhypopituitarism with AVPD; did not resolve with treatment	Initially presented with cavitating lung lesion (resolved); sinusitis (resolved)
2	40 months	Remission induction with prednisolone and RTX (2 × 1 g 2 weeks apart) Remission maintenance with 6 monthly RTX (1 g)	At presentation, cyst-like peripherally enhancing pituitary lesion and thickened pituitary stalk with distortion of the optic chiasm; full resolution of cyst-like component with normal pituitary stalk	Persistent AVDP	Sinusitis and epistaxis
3	12 months	Remission induction with prednisolone and RTX (2 × 1 g 2 weeks apart) Remission maintenance with 6 monthly RTX (1 g)	At presentation, enlarged with peripheral enhancement and lack of central enhancement with the dome of the lesion abutting the optic chiasm without overt compression; full resolution of radiological abnormalities	Panhypopituitarism with AVDP; did not resolve with treatment	Sinusitis and epistaxis; nasal cartilage destruction

*GPA* granulomatosis with polyangiitis, *AVPD* arginine vasopressin deficiency, *RTX* rituximab

All 3 patients continue to have cranial AVPD and remain on DDAVP supplementation. Interestingly, our observations differ from those of Kapoor et al., who found that AVPD resolved in 4 out of 6 patients [3]. In addition, patient 1 and 3 remain on hormone replacement for secondary hypoadrenalism and secondary hypothyroidism.

Patient 2, who had localised pituitary GPA and limited sinus involvement only, remains in full remission with 6-monthly RTX infusions and no longer requires glucocorticoid maintenance therapy. In contrast, patient 1 had GPA for 28 months before presenting with GPA-related pituitary hypophysitis and therefore required a longer treatment period to achieve clinical and radiological responses.

RTX treatment was generally well tolerated and none of our patient reported any significant infections. However, patients experienced significant weight gain while on high-dose glucocorticoid therapy.

## Conclusion

Pituitary GPA may occur at variable times after the original diagnosis of AAV and may be the only manifestation, in absence of any other systemic features when disease is otherwise in apparent remission. A combination of glucocorticoids and RTX is approved for systemic GPA, however, limited data is available for treatment of pituitary GPA. In this case series, the response to high dose steroids and RTX for remission-induction and maintenance has been highly encouraging. RTX was well tolerated without any major side effects. Further clinical studies are required to establish the most effective treatments for pituitary GPA.

## Limitations

Limitations of this current study are relatively poor quality of data from the literature due to the rarity of GPA-related hypophysitis. Larger trials to further study different treatment regimens are therefore needed.

## Data Availability

Not applicable.

## References

[1] Jennette JC, Falk RJ, Bacon PA (2013). 2012 revised international Chapel Hill consensus conference nomenclature of vasculitides. Arthritis Rheum.

[2] Kitching AR, Anders HJ, Basu N (2020). ANCA-associated vasculitis Nat Rev Dis Primers.

[3] Kapoor E, Cartin-Ceba R, Specks U, Leavitt J, Erickson B, Erickson D (2014). Pituitary dysfunction in granulomatosis with polyangiitis: the Mayo Clinic experience. J Clin Endocrinol Metab.

[4] De Parisot A, Puechal X, Langrand C (2015). Pituitary involvement in granulomatosis with polyangiitis: report of 9 patients and review of the literature. Medicine (Baltimore).

[5] Vega-Beyhart A, Medina-Rangel IR, Hinojosa-Azaola A (2020). Pituitary dysfunction in granulomatosis with polyangiitis. Clin Rheumatol.

[6] Baird SM, Pratap U, McLean C, Law CP, Maartens N (2017). Rare presentation of Wegener's granulomatosis in the pituitary gland: case report and literature review. Int J Surg Case Rep.

[7] Hellmich B, Sanchez-Alamo B, Schirmer JH (2023). EULAR recommendations for the management of ANCA-associated vasculitis: 2022 update. Ann Rheum Dis.

[8] Specks U, Merkel PA, Seo P (2013). Efficacy of remission-induction regimens for ANCA-associated vasculitis. N Engl J Med.

[9] Stone JH, Merkel PA, Spiera R (2010). Rituximab versus cyclophosphamide for ANCA-associated vasculitis. N Engl J Med.

[10] Ponte C, Grayson PC, Robson JC (2022). American College of Rheumatology/EULAR classification criteria for giant cell arteritis. Arthritis Rheumatol.

[11] Idolor ON, Guraya A, Muojieje CC (2021). Renal involvement in granulomatosis with polyangiitis increases economic health care burden: insights from the national inpatient sample database. Cureus.

[12] Peters JE, Gupta V, Saeed IT, Offiah C, Jawad ASM (2018). Severe localised granulomatosis with polyangiitis (Wegener's granulomatosis) manifesting with extensive cranial nerve palsies and cranial diabetes insipidus: a case report and literature review. BMC Neurol.

[13] Taylor SC, Clayburgh DR, Rosenbaum JT, Schindler JS (2012). Progression and management of Wegener's granulomatosis in the head and neck. Laryngoscope.

[14] Borner U, Landis BN, Banz Y (2012). Diagnostic value of biopsies in identifying cytoplasmic antineutrophil cytoplasmic antibody-negative localized Wegener's granulomatosis presenting primarily with sinonasal disease. Am J Rhinol Allergy.

[15] Zheng Y, Zhang Y, Cai M, Lai N, Chen Z, Ding M (2018). Central nervous system involvement in ANCA-associated vasculitis: what neurologists need to know. Front Neurol.

[16] Hong E, Shalid A, Gatt D, Deepak S, Bahl A (2021). Primary pituitary granulomatosis with polyangiitis and the role of pituitary biopsy, case report and literature review. Br J Neurosurg.

[17] Rao JK, Weinberger M, Oddone EZ, Allen NB, Landsman P, Feussner JR (1995) The role of antineutrophil cytoplasmic antibody (c-ANCA) testing in the diagnosis of Wegener granulomatosis. A literature review and meta-analysis. Ann Intern Med 123(12):925–32 10.7326/0003-4819-123-12-199512150-00005 7486487

[18] Pakalniskis MG, Berg AD, Policeni BA (2015). The many faces of granulomatosis with polyangiitis: a review of the head and neck imaging manifestations. AJR Am J Roentgenol.

[19] Chung SA, Gorelik M, Langford CA (2021). American college of rheumatology/vasculitis foundation guideline for the management of polyarteritis nodosa. Arthritis Rheumatol.

[20] Rovin BH, Adler SG, Barratt J, Bridoux F, Burdge KA, Chan TM (2021). Kidney disease: improving global outcomes glomerular diseases. Kidney Int.

[21] Mukhtyar C, Lee R, Brown D (2009). Modification and validation of the Birmingham vasculitis activity score (version 3). Ann Rheum Dis.

[22] Guillevin L, Pagnoux C, Karras A (2014). Rituximab versus azathioprine for maintenance in ANCA-associated vasculitis. N Engl J Med.

[23] Yates M, Watts R (2017). ANCA-associated vasculitis. Clin Med (Lond).

[24] Charles P, Terrier B, Perrodeau E (2018). Comparison of individually tailored versus fixed-schedule rituximab regimen to maintain ANCA-associated vasculitis remission: results of a multicentre, randomised controlled, phase III trial (MAINRITSAN2). Ann Rheum Dis.

[25] Jones RB, Furuta S, Tervaert JW (2015). Rituximab versus cyclophosphamide in ANCA-associated renal vasculitis: 2-year results of a randomised trial. Ann Rheum Dis.

[26] McAdoo SP, Medjeral-Thomas N, Gopaluni S (2018). Long-term follow-up of a combined rituximab and cyclophosphamide regimen in renal anti-neutrophil cytoplasm antibody-associated vasculitis. Nephrol Dial Transpl.

[27] Gulati K, Edwards H, Prendecki M (2021). Combination treatment with rituximab, low-dose cyclophosphamide and plasma exchange for severe antineutrophil cytoplasmic antibody-associated vasculitis. Kidney Int.

[28] Cortazar FB, Muhsin SA, Pendergraft WF (2018). Combination therapy with rituximab and cyclophosphamide for remission induction in ANCA vasculitis. Kidney Int Rep.

[29] Gendreitzig P, Honegger J, Quinkler M (2020). Granulomatous hypophysitis causing compression of the internal carotid arteries reversible with azathioprine and rituximab treatment. Pituitary.

[30] Smith RM, Jones RB, Specks U (2023). Rituximab versus azathioprine for maintenance of remission for patients with ANCA-associated vasculitis and relapsing disease: an international randomised controlled trial. Ann Rheum Dis.

[31] Jayne DRW, Merkel PA, Schall TJ, Bekker P, Group AS (2021). Avacopan for the treatment of ANCA-associated vasculitis. N Engl J Med.

